# Violence and Suicide Risk in Sub-Saharan Africa: The Case of Senegal

**DOI:** 10.1192/j.eurpsy.2025.913

**Published:** 2025-08-26

**Authors:** J. A. D. Tine, V. Petit, H. M. Langet

**Affiliations:** 1 Medecine, University Cheikh Anta Diop of Dakar, Dakar, Senegal; 2 CEPED, Université Paris Cité, Paris, France; 3 Swiss Centre for International Health (Digital Health Unit) / Research- IT, Swiss Tropical and Public Health Institute, Bale, Switzerland

## Abstract

**Introduction:**

Assessing violence and suicide risk is a critical preventive action in the fight against suicide.

**Objectives:**

This study aimed to analyze the vulnerability factors associated with violence and suicide risk in the general Senegalese population.

**Methods:**

This was an observational, population-based, national, cross-sectional, descriptive, and analytical survey. Data collection was conducted from July to August 2023 on 496 randomly selected households proportionally distributed according to the demographic weight of different areas. The assessment of suicide risk was derived from the Mini International Neuropsychiatric Interview (MINI) V5. Data were directly entered into ODK, and analysis was performed using R software. Ethical approval was granted by the National Health Research Ethics Committee of Senegal.

**Results:**

A total of 2,174 individuals were surveyed, with a mean age of 41.4±12.2 years. The prevalence of violence in Senegal was 52.76%. The most common forms were psychological violence (47.38%), verbal violence (43.47%), and physical violence (32.84%) (Refer to table I: Distribution by type of violence). Associations of violence were observed, with physical violence associated with psychological violence accounting for 19.59%. Women were more exposed to the different type of violence. Geographic disparities were identified, with the Dakar region emerging as the main hotspot for these forms of violence (refer to image 1: mapping of violence by region of Senegal). The suicide risk was found to be 8.4%, with a high level at 1.66%. Regardless of the type of violence, the suicide risk was elevated; it was 47.6% for sexual violence (refer to image 2: Distribution of Suicide Risk According to the Type of Violence). Unfortunately, recourse to victim assistance was low, with 6.2% of victims receiving psychological support and 10.4% receiving police assistance.

**Table I:** Distribution by type of violence

Nature of violence (N=2174)	Numbers	Percentages (%)	IC 95%
Physical violence	714	32.84	[31 - 35]
Sexual violence	83	3.82	[3.1- 4.7]
Psychological violence	1030	47.38	[45 - 50]
Neglect violence	204	9.38	[8.2 - 11]
Domestic violences	332	15.27	[14 -17]
Verbal violence	945	43.47	[41 - 46]
Domestic harassment	273	12.56	[11 -14]
Domestic assault	184	8.46	[7.3 - 9.7]
All violences	1147	52.76	[51 - 55]

**Image 1:**

**Figure:**
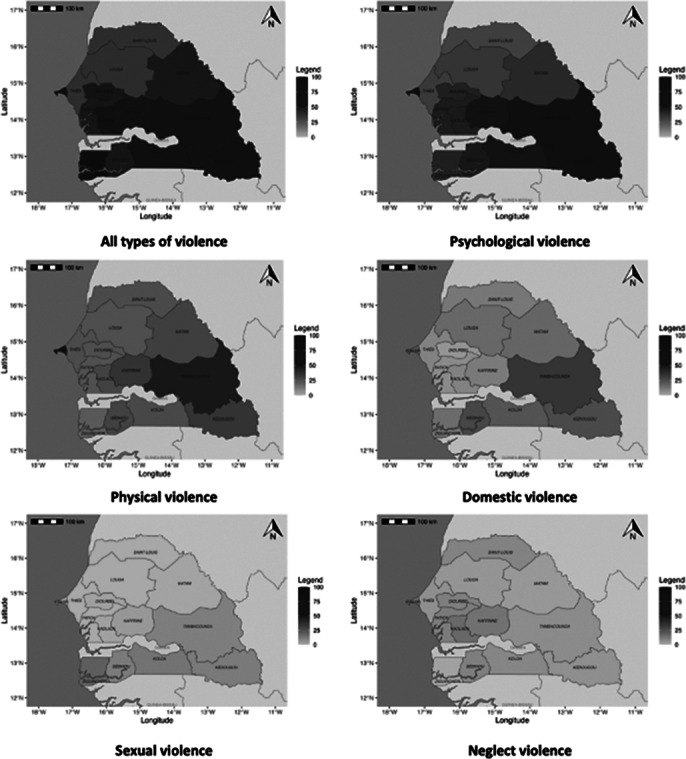

**Image 2:**

**Figure:**
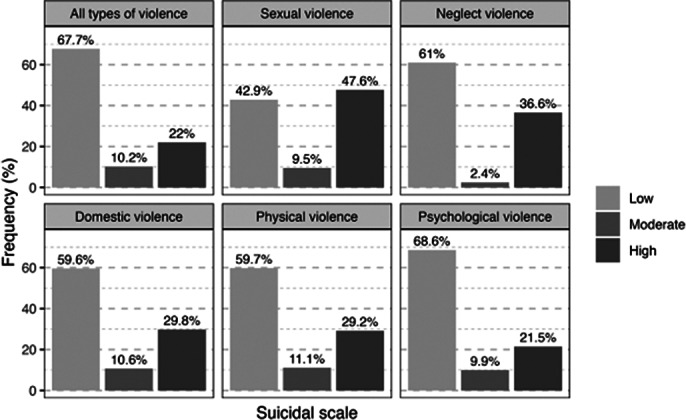

**Conclusions:**

Violence was significant in Senegal, with gaps in administrative and medicopsychological assistance. The suicide risk is high, but suicide remains underreported. It is crucial for Senegal to have a political and health framework with a public health approach centered on vulnerable groups for managing violence and suicide risks.

**Keywords:**

Violence, Suicide risk, Senegal, Africa

**Disclosure of Interest:**

None Declared

